# Supplementary material for: Inhibition of Fatty Acid-Binding Protein 4 Limits High-Fat-Diet-Associated Prostate Tumorigenesis and Progression in TRAMP Mice
Source: Int J Mol Sci. 2025 Oct 31;26(21):10621. doi: 10.3390/ijms262110621 (PMC12607695; doi:10.3390/ijms262110621)
Supplement: Supplementary file 1 [file ijms-26-10621-s001.zip › Supporting information_251010.pdf]

Inhibition of fatty acid-binding protein 4 limits high-fat diet-associated prostate tumorigenesis and progression in TRAMP mice

Supporting information

Table S1. Prostate cancer progression in TRAMP and FABP4$^{-/-}$ TRAMP mice fed different diets.

Eight-week-old TRAMP or FABP4$^{-/-}$ TRAMP male mice were randomly assigned to two different dietary groups: the CE-2 control diet (CD) group and the HF diet group and fed until they reached the desired time points (10, 12, 16, 20, 24, and 30 weeks; 6–12 mice per time subgroup). Thereafter, the mice were sacrificed at each time point. Individual prostate and other tissues were separated, embedded in paraffin, and stained with hematoxylin and eosin (H&E). Prostate lesions and other tumor burdens were histologically analyzed and classified based on the following specifications: (0) normal tissue; (1) hyperplasia; (2) prostatic intraepithelial neoplasia (PIN); (3) primary adenocarcinoma; and (4) adenocarcinoma with invasion and/or metastasis, including venule invasion and lung metastasis.

Table S2. Metabolomic analysis revealed metabolite alterations in TRAMP-HF and FABP4$^{-/-}$ TRAMP-HF mice.

24-week-old mouse prostate samples from the TRAMP-HF (TF) and FABP4$^{-/-}$ TRAMP-HF (TAF) groups were conducted to CE-TOFMS analysis using the Agilent System for the analysis of the charged metabolites (three mice per group).

Figure S1. Gene and protein expression of FABP4 in the urinary organs of FABP4$^{-/-}$ TRAMP mice.

A Genomic DNA was isolated from the urinary organs of eight-week-old TRAMP, FABP4$^{-/-}$, and FABP4$^{-/-}$ TRAMP mice, after which genotyping PCR analysis was performed to detect products positive for *FABP4* and *TRAMP*. The mutant gene expression of *FABP4* was decreased in each urinary organs of FABP4$^{-/-}$ TRAMP mice and FABP4$^{-/-}$ mice. The genomic DNA isolated from wild-type C57BL/6 mice was used as the negative control (NC), whereas DNA derived from FABP4$^{-/-}$ and TRAMP mice were used as the positive control (PC).

Figure S2. Morphological changes in adipocytes from FABP4$^{-/-}$ TRAMP mice.

Histological analysis showed that morphological alterations in periprostatic adipose tissues were smaller and more asymmetry in FABP4$^{-/-}$ TRAMP-HF mice than in TRAMP-HF mice (arrow).

Figure S3. mRNA expression levels of *CXCR2* and *CXCR5* in experimental TRAMP prostate tumors.

mRNA expression levels of *CXCR2* (**A**) and *CXCR5* (**B**) in TRAMP-CD (TC), TRAMP-HF (TF), FABP4$^{-/-}$ TRAMP-HF (TAF), and FABP4$^{-/-}$ TRAMP-CD (TAC) mice at 20 to 30 weeks of age were analyzed.
