# Supplementary figures and images for: Inhibition of Fatty Acid-Binding Protein 4 Limits High-Fat-Diet-Associated Prostate Tumorigenesis and Progression in TRAMP Mice
Source: Int J Mol Sci. 2025 Oct 31;26(21):10621. doi: 10.3390/ijms262110621 (PMC12607695; doi:10.3390/ijms262110621)

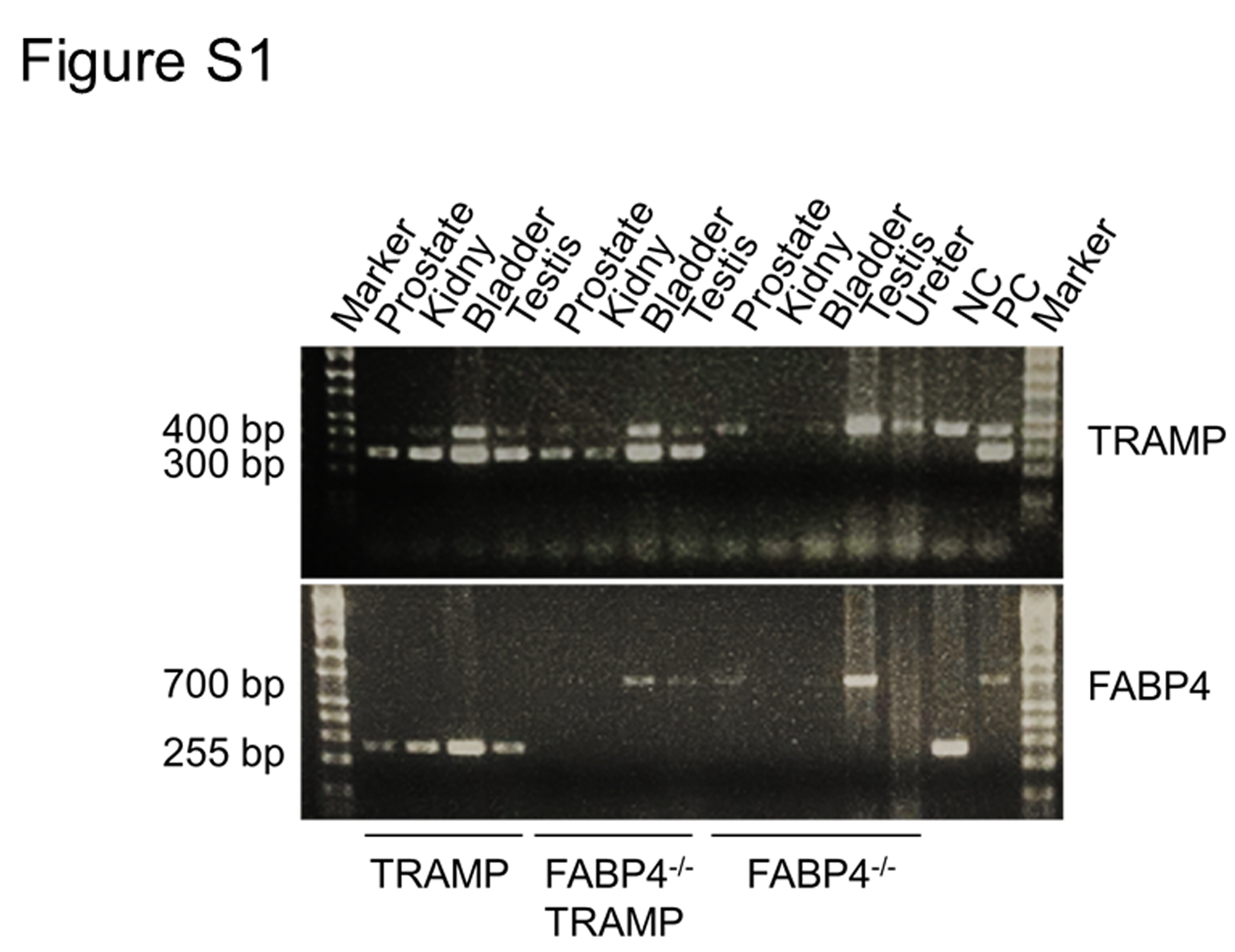

Supplement: Supplementary file 1 [file ijms-26-10621-s001.zip › Figure S1.tif]

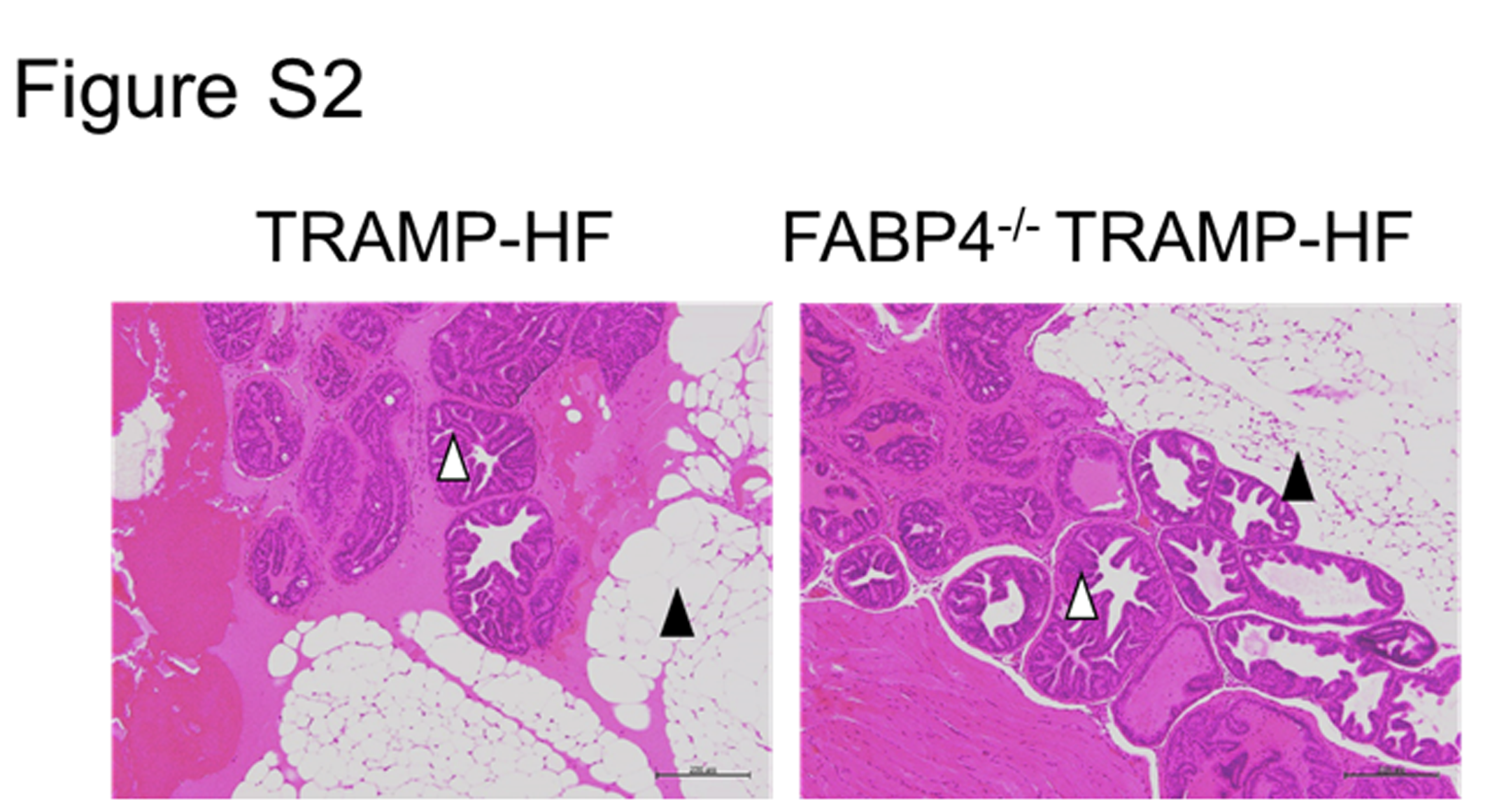

Supplement: Supplementary file 1 [file ijms-26-10621-s001.zip › Figure S2.tif]

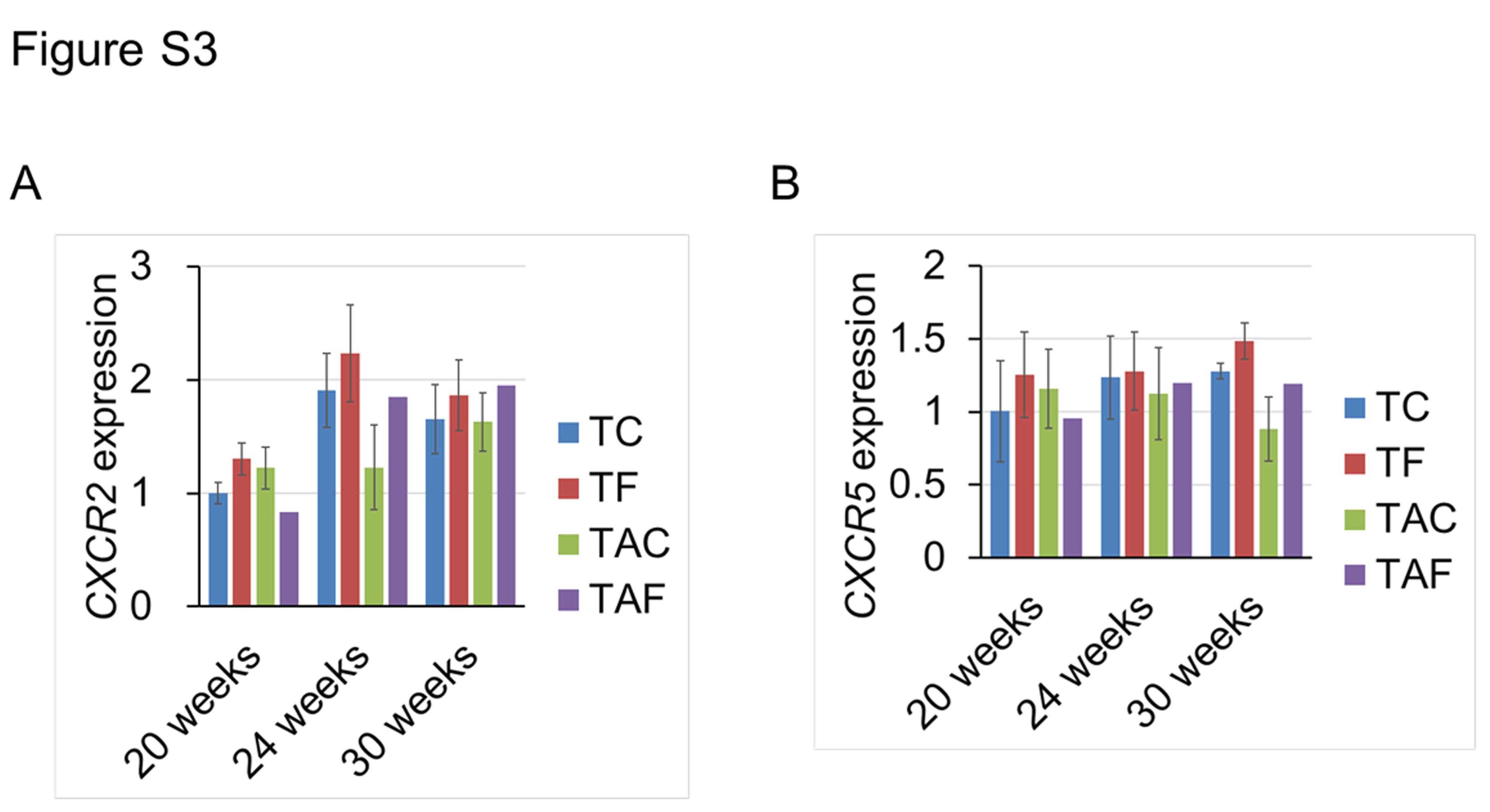

Supplement: Supplementary file 1 [file ijms-26-10621-s001.zip › Figure S3.tif]
